# Anticarcinogenic Effect of Spices Due to Phenolic and Flavonoid Compounds—In Vitro Evaluation on Prostate Cells

**DOI:** 10.3390/molecules22101626

**Published:** 2017-09-28

**Authors:** Zuzana Lackova, Hana Buchtelova, Zaneta Buchtova, Borivoj Klejdus, Zbynek Heger, Martin Brtnicky, Jindrich Kynicky, Ondrej Zitka, Vojtech Adam

**Affiliations:** 1 Department of Chemistry and Biochemistry, Mendel University in Brno, Zemedelska 1, Brno CZ-613 00, Czech Republic; lackova14@seznam.cz (Z.L.); hanabuchtelova@seznam.cz (H.B.); ZanetaBurianova@email.cz (Z.B.); klejdusb@seznam.cz (B.K.); heger@mendelu.cz (Z.H.); zitkao@seznam.cz (O.Z.); 2 Central European Institute of Technology, Brno University of Technology, Technicka 3058/10, Brno CZ-616 00, Czech Republic; martin.brtnicky@mendelu.cz (M.B.); jindrich.kynicky@mendelu.cz (J.K.); 3 Department of Geology and Pedology, Mendel University in Brno, Zemedelska 1, Brno CZ-613 00, Czech Republic

**Keywords:** apigenin, clonogenic assay, 3,4-dihydroxybenzaldehyde, MTT assay, naringenin chalcone, neochlorogenic acid, prostate cancer, scratch test, spices

## Abstract

This study shows the effects of spices, and their phenolic and flavonoid compounds, on prostate cell lines (PNT1A, 22RV1 and PC3). The results of an MTT assay on extracts from eight spices revealed the strongest inhibitory effects were from black pepper and caraway seed extracts. The strongest inhibitory effect on prostatic cells was observed after the application of extracts of spices in concentration of 12.5 mg·mL^−1^. An LC/MS analysis identified that the most abundant phenolic and flavonoid compounds in black pepper are 3,4-dihydroxybenzaldehyde and naringenin chalcone, while the most abundant phenolic and flavonoid compounds in caraway seeds are neochlorogenic acid and apigenin. Using an MTT assay for the phenolic and flavonoid compounds from spices, we identified the IC_50_ value of ~1 mmol·L^−1^ PNT1A. The scratch test demonstrated that the most potent inhibitory effect on PNT1A, 22RV1 and PC3 cells is from the naringenin chalcone contained in black pepper. From the spectrum of compounds assessed, the naringenin chalcone contained in black pepper was identified as the most potent inhibitor of the growth of prostate cells.

## 1. Introduction

At present, the influence of dietary habits and food quality, in terms of substance content, on the development of cancer is being increasingly studied [1]. There are a number of studies pointing to the positive effects of fruit and vegetables, prevalently due to compounds in their contents, such as phenols, flavonoids, vitamins and mineral substances [2,3]. On the other hand, spices have been involved in the human diet for plenty of years, and used as essential additional ingredients for much cooking, and as seasoning. Could they be considered to be one of the major sources of anticarcinogenic compounds because they contain antioxidants and other biologically active molecules? Thus, it is not surprising that numerous species have been studied in the context of their effects on human health. Antibacterial effects are demonstrated for sweet peppers, peppers and caraway seeds [4,5,6]. Marjoram, cinnamon and caraway seeds are also reported to have anti-inflammatory effects [6,7,8]. Cinnamon and caraway seeds are shown to have anticarcinogenic effects [6,7]. Thyme, pepper and oregano are used as antifungals [5,9,10]. The anticarcinogenic effects of spices are attributed to them containing phenolic compounds [11].

Therefore, we focused on the effects of the most prevalent polyphenols on prostate cells, with regards to the fact that the urogenital tract is the most exposed tissue upon which the effect of the chemical substances present in fluids passing through it should have the greatest impact [12]. To date, a number of studies show the anticarcinogenic effects of piperine [13], capsaicin [14] and curcumin [15,16,17] on prostate cancer cells. Of the eight kinds of spice tested in this work, studies on the anticarcinogenic effect on prostate cancer cells have been performed on black pepper only [13,18]. For oregano, marjoram, anise, thyme, sweet pepper, cinnamon and caraway seeds, no studies have yet been published on their effects on prostate cells [7,11,19,20,21,22,23,24].

For our experiments, we directed our attention to phenolic (neochlorogenic acid and 3,4-dihydroxybenzaldehyde) and flavonoid (apigenin and naringenin chalcone) compounds. Apigenin is a flavonoid compound that is abundantly present in fruits and vegetables. Apigenin reduces low density lipoprotein and cholesterol levels; stimulates PPAR-γ; augments the endogenous antioxidants; regulates the death-signaling of reactive oxygen species [25]; regulates inflammatory mediators, including IL-1β and TNF-α [26]; inhibits tumor growth and angiogenesis induced by different cancer cells; and has antiproliferative and antitumor properties in the colon, pancreas and prostate cancer cells [27]. In addition, it was revealed that apigenin can disrupt cancer cell motility by suppressing the focal adhesion kinase/Src signaling [28], which is a key step in the development of tumors and, ultimately, metastasis. Naringenin chalcone is flavonoid compound and its inhibitory effects are demonstrated in U87MG cells [29]. Neochlorogenic acid is a phenolic acid, which exhibits antioxidant and chemopreventive activity in colon and breast cancer, and in U937 leukemia cells; it protects cells from oxidative stress by scavenging reactive oxidative stress (ROS), and suppressing the proliferation of breast and colon cancer cells [30]. The study [31] showed strong inhibition of growth on a breast cancer cell line (MDA-MB-435) and low toxic effect on a normal breast cell line (MCF-10A). The phenolic compound 3,4-dihydroxybenzaldehyde has antioxidant and anti-inflammatory effects: it decreases the proliferation of human breast cancer, and induces apoptosis with inhibition of casein kinase II activity in leukemia cells [32]. However, the potential anticancer mechanisms of phenolic (neochlorogenic acid and 3,4-dihydroxybenzaldehyde) and flavonoid (apigenin and naringenin chalcone) compounds have not been elucidated, and the effects of prostate cancer cells have not been tested so far.

The aim of this experiment was to determine the effect of eight selected spice species on three prostate cell lines. The profile representation of the phenolic and flavonoid compounds of the selected spices was performed by liquid chromatography/mass spectrometry (LC/MS). The most representative phenolic and flavonoid compounds were used for the rest of the final evaluation using an MTT assay, a scratch test and a clonogenic assay.

## 2. Results and Discussion

### 2.1. Cell-Line Proliferative Activity Testing (MTT Assay) for Extract from Eight Spices

**Figure 1:**
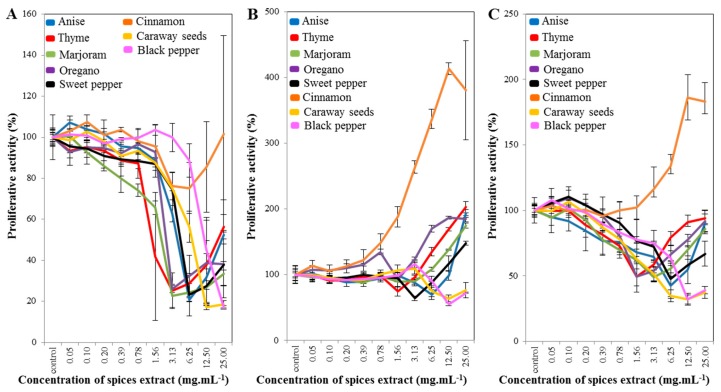
Results of the MTT assay for spice extracts: (**A**) for PNT1A cells; (**B**) for 22RV1 cells; and (**C**) for PC3 cells.

An MTT assay was used to evaluate the cells’ metabolic activity and indicate the cytotoxicity of the tested compounds. Figure 1 shows the results of the effects of the extracts from eight kinds of spice. The results indicate that the best and most stable inhibitory effects were due to treatment with extracts from caraway seeds and black pepper. The strongest inhibitory effect on prostatic cells was observed when we applied a concentration of 12.5 mg·mL^−1^ to all cell lines used. 

If we take a look at the results in greater detail, those of the extracts from anise, thyme, marjoram, oregano and sweet pepper were very similar. A similar effect to those of other extracts for all cell lines used from an extract of cinnamon is shown in Figure 1A. However, difference was observed in the extract from cinnamon (Figure 1B,C), where it was found that the extract from cinnamon was supportive of cell growth here. The inhibitory effect of cinnamon in 29 types of human cancer cells was previously confirmed in vitro, where the vast majority of the antitumorigenic effects of cinnamon extracts could be attributed to cinnamaldehydes [33], the main component of the essential oil. Further, it was described that biologically active substances in cinnamon also cause cell inhibition in prostate cancer cells [33,34]. Other studies describe the inhibitory effect of the selected compound or oil from cinnamon [35,36,37,38], but our study evaluated the effect of cinnamon extract, not the selected components contained in cinnamon, which may explain the deviations from the available literature. The reason for the different effect of cinnamon on prostate cell lines may be that in the aforementioned literature, only the selected cinnamon content has been evaluated, whereas in our study we used a whole-spice extract containing a wide range of substances.

### 2.2. Profiling of the Extracts of the Tested Spices Using LC/MS

Subsequently, an LC/MS analysis was performed to determine which phenolic and flavonoid compounds are contained in selected spices. Determination of the presence and content of phenolic and flavonoid compounds was done using high-performance liquid chromatography with mass detection. The results have been recalculated per 1 g of spice.

**Figure 2:**
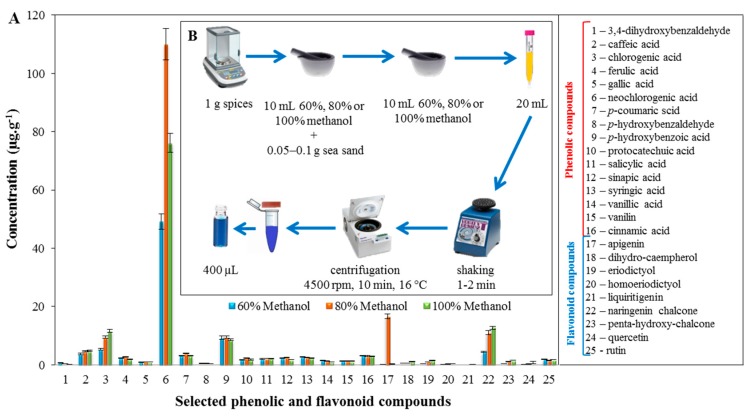
(**A**) Determination of the concentration of selected phenolic and flavonoid compounds in the extract from caraway seeds; (**B**) scheme for preparing the sample for LC/MS analysis.

For the extract from caraway seeds (Figure 2A), the most commonly found phenolic compound was neochlorogenic acid, for which the concentration reached 110 ± 5 μg·g^−1^, with extraction using 80% methanol. The other phenolic compounds determined did not exceed a concentration of 10 μg·g^−1^. Of the flavonoid compounds observed, the highest occurrences recorded in the extract from caraway seeds were for apigenin and naringenin chalcone. The highest concentration of naringenin chalcone had a value of 13 ± 1 μg·g^−1^, with extraction using 100% methanol. A very interesting concentration was found for apigenin when using extraction with 80% methanol, which was 16 ± 1 μg·g^−1^, compared to other types of extraction used. The other flavonoid compounds revealed did not have a concentration greater than 2 μg·g^−1^. Figure 2B describes the preparation of samples for the LC/MS analysis, and more experimental details can be found in Section 3.5.

**Figure 3:**
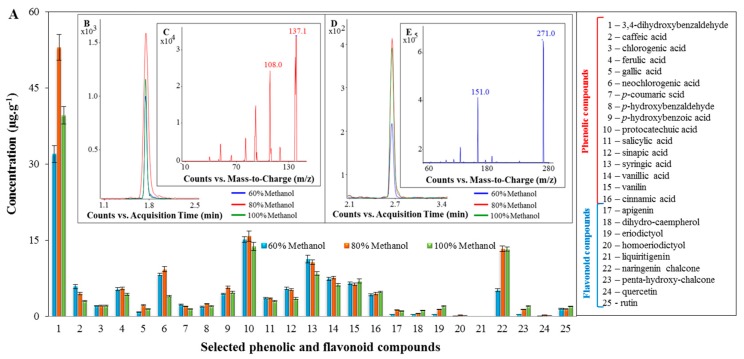
Extract from black pepper: (**A**) determination of concentration of selected phenolic and flavonoid compounds; (**B**) Multiple reaction monitoring (MRM) chromatograms of 3,4-dihydroxybenzaldehyde; (**C**) fragmentation spectrum for 3,4-dihydroxybenzaldehyde (108.0 is the product ion and 173.1 is the precursor ion); (**D**) Multiple reaction monitoring (MRM) chromatograms for naringenin chalcone; and (**E**) fragmentation spectrum for naringenin chalcone (151.0 is the product ion and 271.0 is the precursor ion).

The predominant phenolic compound in the extract from black pepper was 3,4-dihydroxybenzaldehyde. As shown in Figure 3A, the highest concentration was measured using 80% methanol, with the concentration being 55 ± 5 μg·g^−1^. For the other phenolic compounds determined, the concentration was not higher than 16 μg·g^−1^. Contents of protocatechuic acid, caffeic acid, ferulic acid and vanillic acid were also demonstrated in the extract from black pepper in the study [39], despite the different preparation of the sample. The black pepper extract has the highest content of naringenin chalcone compared to other flavonoid compounds. The highest concentration of naringenin chalcone was 13 ± 1 μg·g^−1^, and was measured in the extract obtained using 80% methanol. Comparable results were obtained in the case of using 100% methanol. Using extraction with 60% methanol, the lowest concentration of naringenin chalcone was recorded, that is, 5 ± 1 μg·g^−1^. The results showed that there was less than half the amount of release when using 60% methanol, compared to extractions using 80% and 100% methanol. No significant differences were observed for other flavonoid compounds using extraction with 60%, 80% and 100% methanol. The average concentration of these compounds was 0.8 ± 0.1 μg·g^−1^. In a study by authors Chandra et al. [39], the presence of apigenin and quercetin in black pepper was demonstrated. However, other flavonoid compounds were not investigated here. Figure 3B,D gives a chromatogram of 3,4-dihydroxybenzaldehyde and naringenin chalcone. Figure 3C,E shows a fragmentation spectrum for 3,4-dihydroxybenzaldehyde and naringenin chalcone.

### 2.3. Cell-Line Proliferative Activity Testing (MTT Assay) for Phenolic and Flavonoid Compounds from Spices

**Figure 4:**
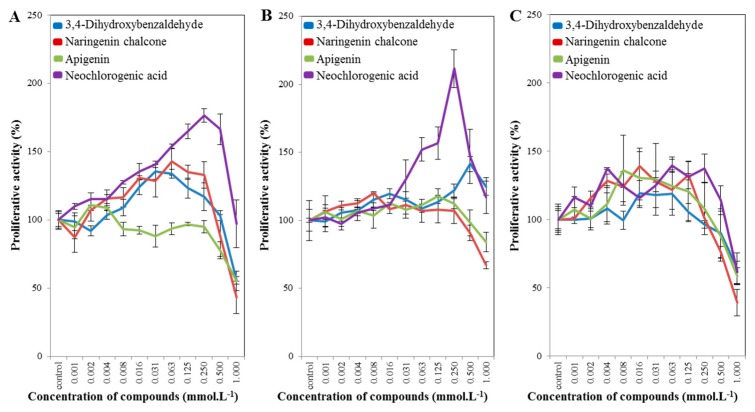
Results of the MTT assay for selected phenolic and flavonoid compounds: (**A**) for PNT1A cells; (**B**) for 22RV1 cells; and (**C**) for PC3 cells.

In the following experiment, we selected caraway seeds and black pepper only, for which we evaluated the presence of selected phenolic and flavonoid compounds (Figure 4). The aim of the MTT assay was to determine the half-maximal inhibitory concentration (IC_50_). According to the obtained results, an IC_50_ value of ~1 mmol·L^−1^ was determined for all cell lines used. The IC_50_ value is too high compared to other studies. However, the aim of this study is not to develop an anticancer drug, but to supplement it and to achieve a better availability of the drug to the organism. The IC_50_ value is 1 mmol·L^−1^ because the substances do not have such toxicity. Studies [40,41,42,43,44] also confirm that flavonoids can only be used to treat cancer as an adjunct to an anticancer drug, not as an anticancer drug alone. Flavonoids thus should serve as a low-dose prevention, not as an acute drug. The identified IC_50_ concentration was then used in further experiments. In PNT1A (Figure 4A), 22RV1 (Figure 4B) and PC3 (Figure 4C) cells, the results were comparable, except for lesser variations in neochlorogenic acid. The most potent inhibitory effect was for the naringenin chalcone for all cell lines used. These results were confirmed in the scratch test, which is described in the results and discussion in Section 2.4. If we compare the clonogenic assay (Table S1, Supplementary Materials) and the MTT assay (Figure 4) for the selected phenolic and flavonoid compounds, we have compliance for the naringenin chalcone and neochlorogenic acid for PC3 cells, and apigenin for PNT1A cells. Studies [27,28] also demonstrate the inhibitory effect of apigenin on prostate cancer cell lines and other cancer cell lines. The inhibitory effect is also demonstrated in naringenin chalcone [29], but not in prostate cancer cell lines. A decreased proliferation of cells is demonstrated when using 3,4-dihydroxybenzaldehyde [32], but not in prostate cancer cell lines. Neochlorogenic acid exhibits chemopreventive activity in colon cancer, and in breast cancer [30], but not in prostate cancer cell lines, where this study is the first one to show these effects.

### 2.4. Wound-Healing Assay (Scratch Test)

**Figure 5:**
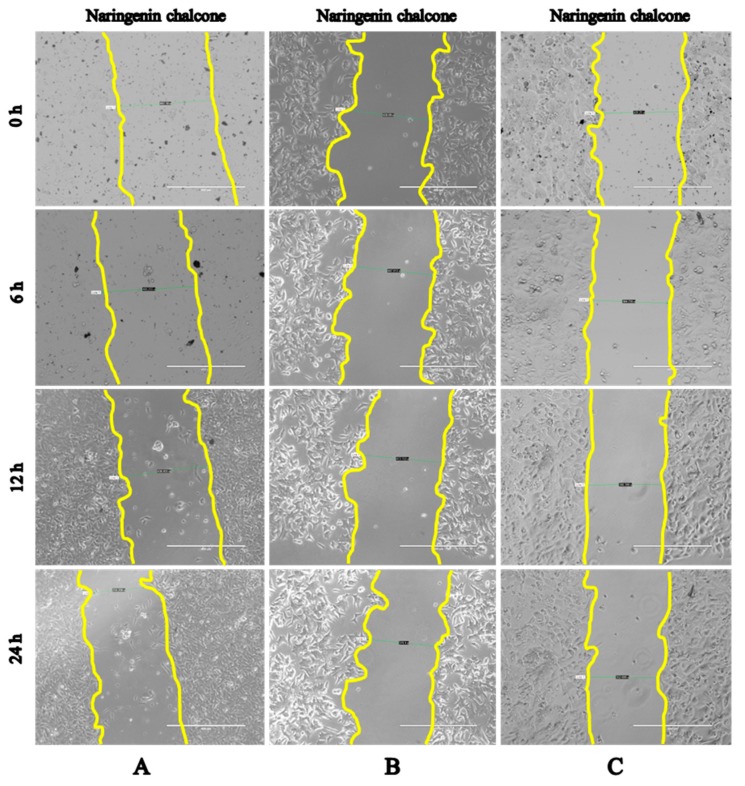
The effect of naringenin chalcone on all three cell lines in the scratch test: (**A**) for PNT1A cells; (**B**) for 22RV1 cells; and (**C**) for PC3 cells.

The scratch test is one of the most widely used, fastest and most effective methods for obtaining the critical loads that are related to the adhesion properties of a coating. A scratch test assesses how power is used in inhibitory compounds for a specific cell. Cells PNT1A, 22RV1 and PC3 showed the strongest inhibitory effects with naringenin chalcone, which had the smallest cell growth over time compared to the control at a time of 0 h (Figure 5A–C and Table 1, Table 2 and Table 3, respectively). The second-strongest inhibitory effect was observed with apigenin in PNT1A, 22RV1 and PC3 cells compared to the control at a time of 0 h (Table 1, Table 2 and Table 3, respectively). The results for the 3,4-dihydroxybenzaldehyde and neochlorogenic acid in PNT1A, 22RV1 and PC3 cells were almost comparable to the control at a time of 0 h (Table 1, Table 2 and Table 3, respectively). The results of the scratch test corroborate the MTT assay results (Figure 4). The microscopy results for other selected phenolic and flavonoid compounds are shown in Figures S1–S3.

Naringenin chalcone is a flavonoid compound that engages in various plant defense roles, due to its antibacterial, antifungal and anti-inflammatory activities and cytotoxicity in carcinoma cells [45,46,47]. Flavonoids occur abundantly in fruits, vegetables, medicinal plants and beverages [43,48]. Flavonoids are present as aglycones, and glycosylated and methylated derivatives. Glycosylated flavonoids are very rich in the human diet. After eating foods containing glycosylated flavonoids, the body hydrolyzes these compounds in the gastrointestinal tract, liberating the aglycones, which are further extensively metabolized by glucuronidation, sulfation or methylation in the small intestine and liver. Flavonoids have poor oral bioavailability, the metabolites predominate in systemic circulation, while the plasma levels of parent flavonoids are very low (below 2 μM or nM range) [40,41,49]. However, the data summarized in the studies [42,44] strongly support the view that flavonoids are promising candidates for the enhancement of oral drug bioavailability and chemoprevention. This is thought to be mainly due to their antioxidant effects, anti-inflammatory properties and ability to modulate metabolism of carcinogens by inhibition of distinct phase 1 metabolic enzymes and activation of phase 2 detoxifying enzymes. Researchers have further demonstrated that methylation of the flavonoids at their free hydroxyl groups or carbon atoms dramatically increases their metabolic stability and enhances membrane transport, leading to facilitated absorption and highly increased oral bioavailability [47].

The mechanism of anticancer action of naringenin chalcone against prostate cancer cells lies in its capability to scavenge free radicals [50], resulting in effects on cell proliferation, inhibition of angiogenesis, inhibition of subcellular signaling and stimulation of DNA repair enzymes [51,52,53,54]. Reactive oxygen species can cause oxidative damage to biological macromolecules including nucleic acids. If cell damage is excessive, cell death or apoptosis occurs. In cells, checkpoint pathways are activated to inhibit progression of cells through the G1 and G2 phases to permit removal of damage and re-entry into the cell cycle. If the DNA damage is not repaired, gene mutations occur at a high rate and can lead to malignant transformation. For repair of oxidatively damaged DNA bases, the base excision repair (BER) pathway is responsible. Removal of the damaged base is a result of increased 8-oxoguanine-DNA glycosylase 1 and apurinic/apyrimidinic endonuclease activities. DNA polymerase β then fills the gap created by the excision of 8-hydroxydeoxyguanosine. Stimulation of the growth of prostate cell carcinoma in prostate cell lines is due to higher production of reactive oxygen species due to the loss of glutathione-S-transferase P1 [50]. The cell-signaling pathways involve cooperation with transcription factors, anti-apoptotic proteins, pro-apoptotic proteins, protein kinases and cell-cycle proteins [55]. For study [55], the IC_50_ value was reported from 200 to 250 µM for naringenin, and growth inhibition was detected. A similar IC_50_ value was recorded in study [56].

## 3. Materials and Methods

### 3.1. Chemicals

The chemicals were purchased from Sigma-Aldrich (St. Louis, MO, USA) in ACS purity, unless noted otherwise. Apigenin standard and neochlorogenic acid standard were purchased from Extrasynthese (Genay, France). Naringenin chalcone standard was purchased from Phytolab (Vestenbergsgreuth, Germany).

### 3.2. Cells

The PNT1A (immortalization of normal, adult, prostatic, epithelial cells), PC3 (androgen-independent) and 22RV1 (androgen-dependent) prostatic cancer cell lines were obtained from the American Type Culture Collection (ATCC) (Manassas, VA, USA). The cells were cultured in a complete RPMI-1640 medium (Hyclone, Waltham, MA, USA) with 10% fetal bovine serum (FBS) (Hyclone, Waltham, MA, USA), supplemented with penicillin and streptomycin (0.1 mg·mL^−1^) at 37 °C and 5% CO_2_ in a humidified incubator.

### 3.3. Preparation of the Spice Samples for Cell-Line Proliferative Activity Testing (MTT Assay)

In this experiment, eight kinds of spice (marjoram, sweet pepper, black pepper, caraway seeds, anise, thyme, cinnamon and oregano) were used. To determine the phenolic and flavonoid compounds from the spices, extraction with 80% (anise, black pepper and caraway seeds) or 100% (thyme, marjoram, sweet pepper, cinnamon and oregano) methanol was used.

1 g sample was weighed for each of eight kinds of spice (Analytical Weight EP 240A, Precisa, Stare Mesto, Czech Republic). The samples of the eight kinds of spice were homogenized in a friction bowl with 10 mL of 80% or 100% methanol, and 0.05 to 0.1 g of sea sand (until evaporation). The homogenization was repeated once more. After the homogenization, the samples were vortexed (Vortex Mixers, VELP Scientifica, Usmate Velate MB, Italy) for 1–2 min, and centrifuged at 4500 rpm and 16 °C for 10 min (Centrifuge Z326K, Hermle, Gosheim, Germany). Subsequently, each sample was filtered through a filter (LUT Syringe Filters Nylon, LABICOM s.r.o., Olomouc, Czech Republic). Samples of the extracts of eight kinds of spice were pipetted (2 mL) and concentrated by nitrogen evaporation at 60 °C.

### 3.4. Cell-Line Proliferative Activity Testing (MTT Assay)

For the extracts from eight kinds of spice, and for the phenolic and flavonoid compounds from the spices, the treatment was initiated after the cells reached ~60–80% confluence. The cells were then harvested, washed four times with phosphate-buffered saline (PBS) (pH 7.4), and counted using the Countess II FL Automated Cell Counter (Life Technologies, Carlsbad, CA, USA). The cells’ proliferative activity was estimated using the MTT assay. Briefly, the suspension of 5000 cells in 50 µL medium was added to each well in the microtiter plates (E-plates 96) used, followed by incubation for 24 h at 37 °C with 5% CO_2_ to ensure cell growth. A volume of 50 µL of the medium containing an extract from eight kinds of spice, and phenolic and flavonoid compounds from the conjugated spices, was added to the cells. To determine the effects on cell proliferative activity, the extract from eight kinds of spice (at a concentration of 0.05–25.00 mg·mL^−1^), and phenolic and flavonoid compounds from spices (at a concentration of 0.001–1 mmol·L^−1^) were employed. The treated cells were incubated for 24 h at 37 °C with 5% CO_2_. In addition, 10 µL of 3-(4,5-dimethylthiazol-2-yl)-2,5-diphenyltetrazolium bromide (MTT (5 mg·mL^−1^ in PBS)) was added to the cells and the mixture was incubated for 4 h at 37 °C. The MTT-containing medium was replaced by 100 µL of 99.9% dimethyl sulfoxide to dissolve MTT–formazan crystals and, after 5 min incubation, the absorbance of the samples was determined at 570 nm (VersaMax Microplate Reader, Molecular Devices, Sunnyvale, CA, USA). The experiments were performed in triplicate.

### 3.5. Preparation of the Samples of Spices and the Analysis of Spice Extracts Using LC/MS

To determine the phenolic and flavonoid compounds of the spices, extraction with 80% (anise, black pepper and caraway seeds) or 100% (thyme, marjoram, sweet pepper, cinnamon and oregano) methanol was used. 

1 g sample was weighed for each of eight kinds of spice (Analytical Weight EP 240A, Precisa, Czech Republic). The samples of the eight kinds of spice were homogenized in a friction bowl with 10 mL of 80% or 100% methanol, and 0.05 to 0.1 g of sea sand (until evaporation). The homogenization was repeated once more. After homogenization, the samples were vortexed (Vortex Mixers, VELP Scientifica, Usmate Velate MB, Italy) for 1–2 min, and centrifuged at 4500 rpm and 16 °C for 10 min (Centrifuge Z326K, Hermle, Gosheim, Germany). Subsequently, each sample was filtered through a filter (LUT Syringe Filters Nylon, LABICOM s.r.o., Olomouc, Czech Republic). Samples of the extracts of the eight kinds of spice were pipetted (400 µL) and analyzed using LC/MS.

To determine the selected phenolic and flavonoid compounds, a high-performance liquid chromatograph (HPLC Agilent 1200 Series) with a diode array detector and a triple quadrupole mass detector (6460 Triple Quad) LC/MS was used. For the separation of the phenolic and flavonoid compounds, a column, Zorbax EC 18 of dimensions 50 mm × 3.0 mm and a particle size of 2.7 μm, was used. The column was held at 45 °C. Mobile phase A consisted of 100% methanol, and mobile phase B was 0.2% acetic acid. The flow rate of the mobile phase was 0.6 mL·min^−1^. The compounds were eluted with a linear upward gradient: 0.00 min (85% B), 0.17 min (85% B), 0.50 min (75% B), 1.70 min (70% B), 4.00 min (70% B), and 6.00 min (85% B). The triple quadrupole mass detector was operated in negative mode. The gas (nitrogen) temperature was 300 °C, the gas flow rate was set to 12 L·min^−1^, the pressure nebulizer had a value of 45 psi, the temperature of the focusing gas was 250 °C, the flow rate of the focusing gas was set at 11 L·min^−1^, and the voltage on the capillary tube amounted to 3500 V.

### 3.6. Wound-Healing Assay (Scratch Test)

The treatment was initiated after the cells reached ~100% confluence. The cells were then harvested, washed four times with PBS (pH 7.4), and counted using the Countess II FL Automated Cell Counter (Life Technologies, Carlsbad, CA, USA). Briefly, the suspension of 10^5^ cells in the medium was added to each well in the microtiter plates (E-plates 6) used, followed by incubation after reaching 100% confluence at 37 °C with 5% CO_2_. After 24 h of serum starvation, a wound was made in the cell monolayer using a 200 μL pipette tip. The cells were washed using PBS, and treated with 2 mL of the medium containing the phenolic and flavonoid compounds from the spices. To determine the effects on cell proliferative activity, the phenolic and flavonoid compounds from the conjugated spices were added to the cells, and a concentration of 1 mmol·L^−1^ was employed. Images at a 400 µm magnification were obtained using an EVOS FL Auto Cell Imaging system (ThermoFisher Scientific, Waltham, MA, USA) at 0, 6, 12 and 24 h of treatment. Within each wound, we analyzed five distance measurements using the EVOS FL Auto software (version 1.7). The experiments were performed in duplicate and each microtiter plate measured five times.

### 3.7. Clonogenic Assay

The cells were then harvested, washed four times with PBS (pH 7.4), and counted using the Countess II FL Automated Cell Counter (Life Technologies, Carlsbad, CA, USA). Briefly, the suspension of 10^3^ cells in the medium was added to each well in the microtiter plates (E-plates 6) used, followed by incubation for 24 h at 37 °C with 5% CO_2_. After 24 h, the medium was removed, and the cells treated with 2 mL of the medium containing the phenolic and flavonoid compounds from the spices. To determine the effects on cell proliferative activity, the phenolic and flavonoid compounds from the conjugated spices were added to the cells, and a concentration of 1 mmol·L^−1^ was employed. This was followed by incubation for 24 h at 37 °C with 5% CO_2_, then a medium change and incubation for 9–14 days at 37 °C with 5% CO_2_. The medium was removed and the cells were washed with PBS and fixation was completed with methanol:acetic acid (3:1) for 5 min. After fixation, the cells were colored with 0.5% crystal violet in methanol for 15 min. The cells were washed with Milli-Q water. Images were obtained using a Canon EOS 650D (Canon, Ota, Japan). The experiments were performed in duplicate.

## 4. Conclusions

The MTT assay we performed on the extracts from eight spices revealed the strongest inhibitory effects of caraway seeds and black pepper on the tested cell lines. From the black pepper and caraway seeds, we analyzed selected phenolic and flavonoid compounds using LC/MS. The most represented phenolic and flavonoid compounds were, in caraway seeds, neochlorogenic acid, and in black pepper, 3,4-dihydroxybenzaldehyde and naringenin chalcone. The results of the MTT assay for these compounds determined the value of IC_50_ (1 mmol·L^−1^ for the PNT1A, 22RV1 and PC3 cells). The most potent inhibitory effect on the PNT1A, 22RV1 and PC3 cells was from the naringenin chalcone (concentration 1 mmol·L^−1^) contained in black pepper, found using the scratch test. The inhibitory effect of naringenin chalcone was confirmed in other studies, but in cells other than prostatic cells. The results obtained serve as a pilot study for further experiments, where other cells lines and/or potency of some identified biologically active molecules will be tested.

## Figures and Tables

**Table 1:** Results of the scratch test for PNT1A cells.

Compounds	0 h		6 h		12 h		24 h	
	Measured Value *	%	Measured Value *	%	Measured Value *	%	Measured Value *	%
Control	518.12 ± 6.58	100	443.24 ± 6.52	86	377.50 ± 2.13	73	80.39 ± 20.27	16
3,4-Dihydroxybenzaldehyde	505.10 ± 5.10	100	441.11 ± 6.82	97	398.04 ± 7.94	79	147.72 ± 2.92	29
Naringenin chalcone	492.72 ± 5.38	100	446.06 ± 8.28	91	431.77 ± 7.32	88	269.33 ± 3.78	55
Apigenin	490.69 ± 3.78	100	397.23 ± 6.59	81	360.21 ± 6.22	73	176.18 ± 9.94	36
Neochlorogenic acid	519.38 ± 7.00	100	436.78 ± 7.17	84	303.23 ± 6.49	58	71.92 ± 2.37	14

* The average of the five measurements.

**Table 2:** Results of the scratch test for 22RV1 cells.

Compounds	0 h		6 h		12 h		24 h	
	Measured Value *	%	Measured Value *	%	Measured Value *	%	Measured Value *	%
Control	474.72 ± 7.77	100	420.75 ± 9.88	89	371.84 ± 11.27	78	140.11 ± 10.24	30
3,4-Dihydroxybenzaldehyde	406.92 ± 12.63	100	349.11 ± 13.20	86	304.66 ± 8.34	75	152.38 ± 11.25	37
Naringenin chalcone	461.85 ± 9.27	100	436.98 ± 10.68	95	411.39 ± 8.32	89	398.17 ± 11.82	86
Apigenin	480.67 ± 5.35	100	415.30 ± 6.16	86	368.72 ± 8.68	77	300.77 ± 8.78	63
Neochlorogenic acid	365.15 ± 4.35	100	318.30 ± 7.54	87	300.08 ± 6.22	82	139.02 ± 15.20	38

* The average of the five measurements.

**Table 3:** Results of the scratch test for PC3 cells.

	0 h		6 h		12 h		24 h	
Compounds	Measured Value *	%	Measured Value *	%	Measured Value *	%	Measured Value *	%
Control	494.87 ± 7.25	100	436.01 ± 2.03	88	369.92 ± 7.54	75	136.69 ± 22.52	28
3,4-Dihydroxybenzaldehyde	477.30 ± 3.73	100	408.49 ± 2.33	86	373.09 ± 3.78	78	102.62 ± 8.73	22
Naringenin chalcone	444.85 ± 7.66	100	410.23 ± 7.47	92	398.44 ± 3.23	90	360.25 ± 6.42	81
Apigenin	398.55 ± 8.46	100	370.29 ± 6.75	93	341.14 ± 8.87	86	270.30 ± 9.43	68
Neochlorogenic acid	448.47 ± 6.80	100	393.60 ± 8.87	88	356.98 ± 8.52	80	136.54 ± 10.86	30

* The average of the five measurements.

## Supplementary Materials

Supplementary Materials are available online, Figures S1–S3 and Table S1.
