# Supplementary material for: Anticarcinogenic Effect of Spices Due to Phenolic and Flavonoid Compounds—In Vitro Evaluation on Prostate Cells
Source: Molecules. 2017 Sep 28;22(10):1626. doi: 10.3390/molecules22101626 (PMC6151579; doi:10.3390/molecules22101626)
Supplement: Supplementary file 1 [file molecules-22-01626-s001.pdf]

molecules

Article, Supplementary Information

Anticarcinogenic Effect of Spices Due to Phenolic and Flavonoid Compounds—in Vitro Evaluation on Prostate Cells

Zuzana Lackova [1,2], Hana Buchtelova [1,2], Zaneta Buchtova [1], Borivoj Klejdus [1], Zbynek Heger [1,2], Martin Brtnicky [2,3], Jindrich Kynicky [2,3], Ondrej Zitka [1,2] and Vojtech Adam [1,2,*]

[1] Department of Chemistry and Biochemistry, Mendel University in Brno, Zemedelska 1, Brno CZ-613 00, Czech Republic; lackova14@seznam.cz (Z.L.); hanabuchtelova@seznam.cz (H.B.); ZanetaBurianova@email.cz (Z.B.); klejdusb@seznam.cz (B.K.); heger@mendelu.cz (Z.H.); zitkao@seznam.cz (O.Z.)

[2] Central European Institute of Technology, Brno University of Technology, Technicka 3058/10, Brno CZ-616 00, Czech Republic; martin.brtnicky@mendelu.cz (M.B.); jindrich.kynicky@mendelu.cz (J.K.)

[3] Department of Geology and Pedology, Mendel University in Brno, Zemedelska 1, Brno CZ-613 00, Czech Republic

* Correspondence: vojtech.adam@mendelu.cz; Tel.: +420-5-4513-3350; Fax: +420-5-4521-2044

Wound healing assay (scratch test)

In section 2.3., the effect of naringenin chalcone is described in the results, which acted as the most potent inhibitor for the PNT1AA, 22RV1, and PC3 cells. The results of the other tested phenolic and flavonoid compounds are shown here. Figure 1S gives the results for PNT1AA cells. A similar inhibitory effect was seen in 3,4-dihydroxybenzaldehyde and apigenin for the PNT1AA cells compared to a time of 0 h. No inhibitory effect was observed in the neochlorogenic acid compared to a time of 0 h.

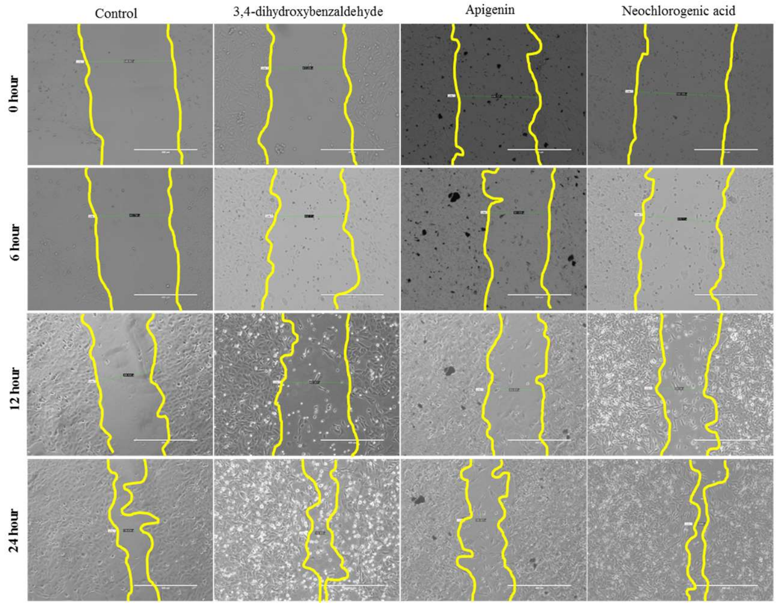

Figure 1S. Results of the scratch test for the PNT1AA cells.

According to the results for the 22RV1 cells, Figure 2S reveals the second strongest inhibitor is apigenin compared to a time of 0 h. A comparable effect was detected in 3,4-dihydroxybenzaldehyde and neochlorogenic acid compared to the control and to a time of 24 h.

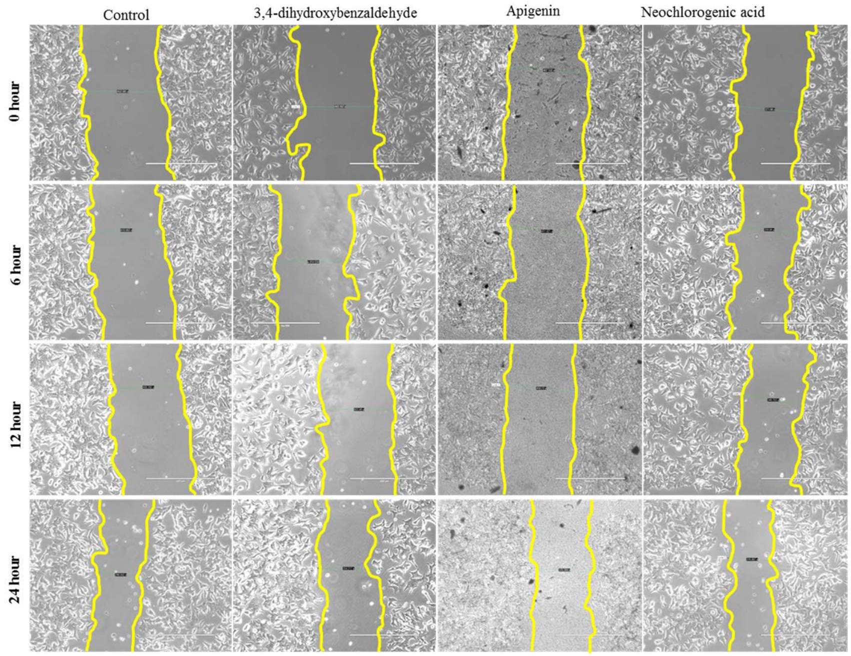

Figure 2S. Results of the scratch test for the 22RV1 cells.

Similar results to those for PNT1AA and 22RV1 cells were also evaluated in the PC3 cells (Figure 3S). The second strongest inhibitor was apigenin compared to the control. A similar inhibitory effect was seen in 3,4-dihydroxybenzaldehyde and neochlorogenic acid for the PNT1AA cells compared to a time of 0 h.

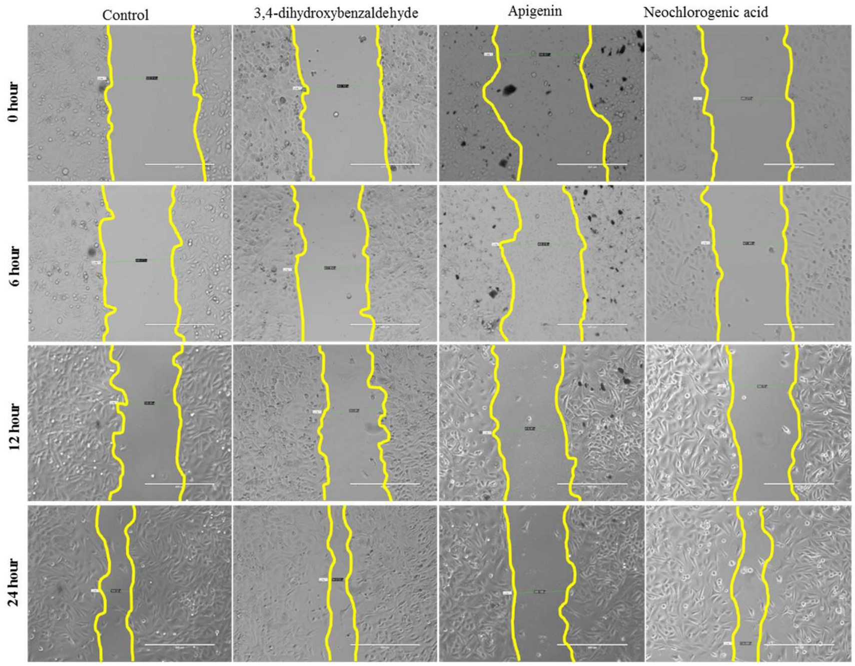

Figure 3S. Results of Scratch test for PC3 cells.

Clonogenic assay

A clonogenic assay assesses the number of colonies growing after treatment with the test compound (Table 1S). In all cell lines, the number of colonies was reduced after treatment with the

compounds as compared to the control. For the 22RV1 and PC3 cells, the lowest number of colonies was observed with neochlorogenic acid, and for PNT1AA cells the lowest number occurred with apigenin. Conversely, the highest number of colonies compared with the control was observed in the 22RV1 cells with 3,4-dihydroxybenzaldehyde, and in the PNT1AA and PC3 cells for naringenin chalcone. The clonogenic assay serves only as a supplement to the MTT assay; however, the MTT assay has a higher displacement value. If we compare the MTT assay (Figure 4) and the clonogenic assay for selected phenolic and flavonoid compounds, we have similar results only for naringenin chalcone and neochlorogenic acid for the PC3 cells, and for apigenin for the PNT1AA cells.

Table 1S. Results of Clonogenic assay for PNT1AA, 22RV1, and PC3 cells.

	**PNT1A cells**		**.22RV1 cells**		**PC3 cells**	
**Compounds**	**Number of colonies***	**%**	**Number of colonies***	**%**	**Number of colonies***	**%**
Control	21.5	100	132.5	100	164.5	100
3,4-dihydroxybenzaldehyde	2.5	12	44.5	34	40.5	25
Naringenin chalcone	19.0	88	31.0	23	46.5	28
Apigenin	1.5	7	34.0	26	31.5	19
Neochlorogenic acid	3.0	14	2.5	2	24.5	15

* the average of two measured values
